# The Deadly Policeman’s Club and the Brutal Clubber

**Published:** 1892-12

**Authors:** 


### Selections and Abstracts.

THE DEADLY POLICEMAN'S CLUB AND THE BRUTAL CLUBBER. —In the October number of the *Alienist and Neurologist*, we find the following sensible editorial:

Why does modern civilization permit policemen to strike on the head with their deadly clubs? The club of the policeman is a relic of the barbarous ages and the man who would use it violently except in the plainest self-defense of imperilled life is a barbarian brute even though he may have been taught to pray to the Holy Virgin and have St. Patrick for his patron Saint. There are Christian as well as heathen brutes.

Why should a policeman strike a fellow-man with such a deadly weapon on the head? A resisting prisoner is just as easily overcome by blows on the arms and the victim's future is not thereby imperilled. The risk of insanity and death are not great from a broken arm.

The damage done by the club is not fully known to the public because its victims belong to the defenseless and friendless class, who go without sympathetic following to the public hospitals, asylums, and to Potter's field, like a poor big-headed Willie, or Hartnett, of St. Louis, now on trial in a distant city for murder done under the influence of insanity caused by a policeman's club on his head in St. Louis, several years ago while drunk.

Many of the cases of insanity attributed to drunkenness in pauper asylums are the results of policemen's club violence on the head.

Is there no regulation prohibiting striking on the head? Is there no law on the subject? If not, there should be both without delay.

Aside from the humanitarian aspect of the subject, taxpayers do not wish to have brain diseases and lunatics to be cared for at public expense, made through the brutal use of the club.

But humanity demands that the use of the club should be restricted. The club is brutal anyway. It makes both policeman and criminal brutal, and the man who would use it on an unoffending prisoner or upon an offending or offensive one, about the head, except in case of the direct necessity of defending his own life, is "a wretch whom it were base flattery to call a coward."

Science, especially psychological science, and humanity protest against the brutalism of past ages. Let the club be abolished and the clubber suppressed.

